# A biomechanical comparison of fibular strut and femoral head allograft for augmented locking plate fixation in three-part proximal humerus fractures

**DOI:** 10.1016/j.jseint.2026.101625

**Published:** 2026-01-14

**Authors:** Stijn R.J. Mennes, Igor J. Shirinskiy, Charmaine E.M. Kool, Eric D. Tutuhatunewa, Ronald L.A.W. Bleys, Tjarco D.W. Alta, Michel P.J. van den Bekerom, Laura M. Kok

**Affiliations:** a Shoulder and Elbow Unit, Department of Orthopaedic Surgery, OLVG, Amsterdam, The Netherlands; b Department of Orthopaedic and Trauma Surgery, Flinders Medical Centre, Adelaide, Australia; c Faculty of Behavioural and Movement Sciences, Department of Human Movement Sciences, Vrije Universiteit Amsterdam, Amsterdam Movement Sciences, Amsterdam, The Netherlands; d Department of Orthopaedic Surgery, Tergooi MC, Hilversum, The Netherlands; e Department of Anatomy, University Medical Center Utrecht, Utrecht, The Netherlands; f Department of Orthopaedics and Traumatology, Spaarne Gasthuis, Haarlem/Hoofddorp, The Netherlands

**Keywords:** Proximal humerus fracture, Augmentation, Locking plate, Fixation, Cortical, Cancellous, Fibula, Femoral head

## Abstract

**Background:**

It is not clear whether cortical fibular allografts or cancellous femoral allografts provide similar stability for augmentation of locking plate fixation of proximal humerus fractures. Therefore, this study aimed to assess and compare biomechanical properties of augmented locking plate fixation with 1) a cortical fibular allograft and 2) a cancellous femoral allograft.

**Methods:**

Twenty-two fresh frozen humeri were randomly allocated to undergo unstable three-part fracture creation, locking plate fixation, and augmentation with cortical fibular or cancellous femoral allografts. All constructs were tested with cyclic loading (5 N to 532.5 N) for 1000 cycles at 1 Hz. Subsequently, constructs were loaded to 1700 N. Loss of humeral head height (HHH), ultimate failure loads, mode of failure, and stiffness were assessed.

**Results:**

Twenty humeri were included in the analysis. No failure occurred during cyclic loading. During load-to-failure testing, four (40.0%) fibula-augmented constructs and six (60.0%) femoral-augmented constructs failed. During cyclic loading, median loss of HHH was 2.00 (interquartile range [IQR], 1.12-3.50) for fibular grafts and 2.75 (IQR, 2.00-3.00) femoral grafts (*P* = .40). Median loss of HHH during failure tests was 9.50 (IQR, 7.88-15.50) for fibular grafts and 9.50 (IQR, 8.50-10.90) for femoral grafts (*P* = .68). The stiffness for fibular grafts was 174 (IQR, 106-186) and 157 for femoral grafts (IQR, 134-192) (*P* = .97).

**Conclusion:**

There is no significant difference in biomechanical stability between cortical fibular allograft augmentation and cancellous hip allograft augmentation for locking plate fixation in proximal humerus fractures. Both techniques may equally prevent loss of reduction and early screw protrusion. Cancellous femoral allografts may yield advantages considering costs, availability, and future revision surgery with arthroplasty when compared to cortical fibular allografts.

Locking plate fixation for proximal humerus fractures (PHFs) is associated with high complication rates, especially in osteoporotic fractures.^29^^,^^33^ For instance, a study from the Mayo Clinic reported a complication rate of 44% after plate fixation in patients aged 60 years or older, tending to increase with age.^3^ The most frequently reported complications comprise intra-articular screw penetration and loss of reduction, leading to avascular necrosis, nonunion, and functional impairment.^34^ High complication rates in older patients may be due to loss of bone density, resulting in bone void and less mechanical support of the humeral medial column due to comminution. This lack of medial support may result in early loss of reduction and poor clinical outcomes after plate fixation.^14^^,^^17^^,^^20^

To account for this insufficient medial support, several alternative strategies have shown promise. Intramedullary nailing provides similar rotational stability as locking plates during regular motion,^12^ surgery is less invasive, and varus collapse and (secondary) screw protrusion are observed less frequently.^39^ However, outcomes become less predictable when fracture complexity increases.^37^ Moreover, augmentation with an additional medial plate^31^ or cement may result in greater stability.^18^ Recently, augmentation of plate fixation with bone grafts has shown promising results in decreasing complications in plate fixation. Cortical fibular grafts improve medial support, enhancing locking plate stability and reducing the risk of failure.^19^^,^^24^ A 2022 review reported good clinical and radiological outcomes in patients treated with cortical fibular allograft augmentation for PHFs.^9^ Furthermore, biomechanical studies have shown greater stiffness and maximal loads to failure compared to locking plates without bone grafts.^2^^,^^7^^,^^15^ However, cortical grafts such as fibular grafts will complicate revision surgery (eg, arthroplasty) due to bone fusion of the graft and obliteration of the intramedullary canal after graft removal.^1^^,^^22^ This obliteration increases the risk of humeral shaft fractures and false routes during stem implantation.

Therefore, Euler et al suggested a different cancellous (allogenic femoral or humeral head) allograft that utilizes more contact area between the graft and proximal humerus, potentially enhancing stability.^10^ In addition, cancellous grafts pose fewer challenges during revision surgery. The authors reported satisfactory functional and radiological outcomes using this method. Dankl et al further explored this method by testing the mushroom-shaped (femoral) allograft biomechanically. They found a greater stiffness and load to failure in combination with a locking plate compared to a locking plate without graft.^8^ However, to our knowledge, stiffness and load to failure of cortical fibular and cancellous femoral allografts have never been compared directly.

Therefore, this biomechanical study aims to assess and compare displacement, stiffness, and load to failure of augmented locking plate fixation with 1) a cortical fibular allograft and 2) a cancellous femoral allograft in PHFs.

## Materials and methods

### Specimen preparation

Twenty-two fresh-frozen human shoulders were used to compare load to failure and stiffness of the allografts in combination with a locking plate. All specimens, humeri, and allografts were stored at −20°C and thawed at room temperature before use. The soft tissues, including the rotator cuff, biceps tendon, glenohumeral ligaments, and deltoid insertion, were removed completely from all humeri. All shoulders were randomly assigned to two groups to minimize the effects of differences between left and right shoulders. The first group of eleven shoulders received locking plate fixation with a cortical fibular allograft; the second group of eleven shoulders received locking plate fixation with a cancellous femoral head allograft. All specimens were obtained through a donation program via the Department of Anatomy of the University Medical Centre Utrecht. Written informed consent during life was given by the body donors, allowing the use of their entire bodies for scientific and educational purposes.

### Fracture simulation and surgical techniques

**Figure 1:**
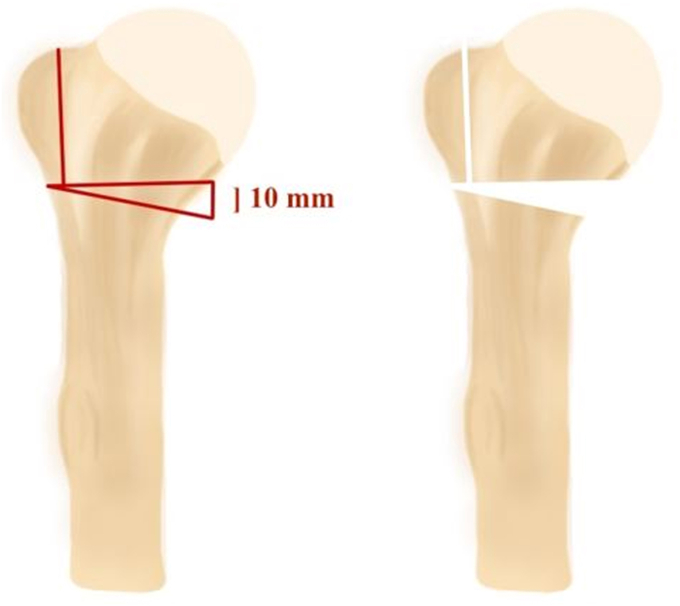
Schematic overview of the unstable three-part proximal humerus fracture simulation.

**Figure 2:**
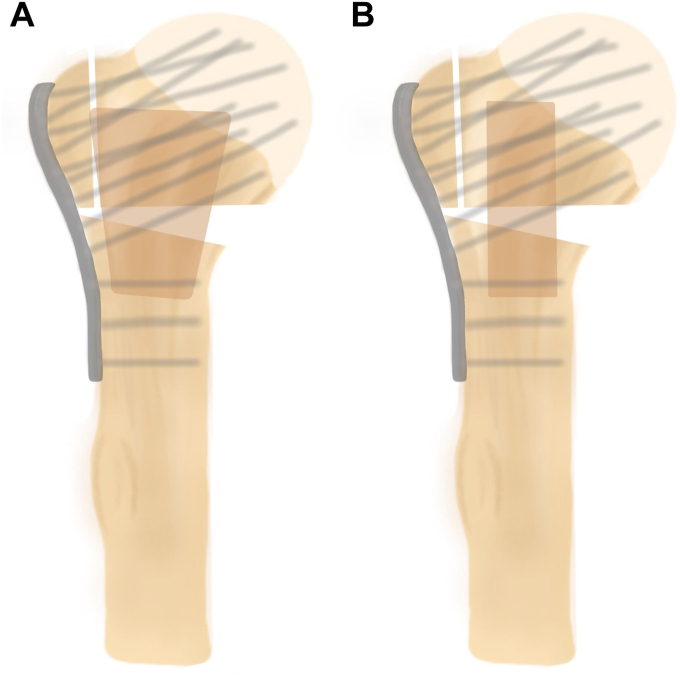
(**A**) Schematic overview of locking plate fixation augmented with a cancellous femoral allograft. (**B**) Schematic overview of locking plate fixation augmented with a cortical fibular allograft.

An unstable three-part fracture was simulated by performing an osteotomy of the greater tubercle at the medial footprint and a V-shaped osteotomy of the surgical neck, simulating medial comminution using a cutting guide (Fig. 1). Fracture lines were marked on each shoulder. The osteotomy was performed using an oscillating saw. For femoral head allograft preparation, all cartilage and cortical bone was removed and the graft was prepared with a shaping guide to fit in the fractured shoulder^8^^,^^10^ (Fig. 2, *A*). All cancellous grafts were 4 cm in length with diameters of 2.5 cm proximally and 1 cm distally. The grafts were then shaped mushroom-like to fit perfectly into the humeral head and shaft, aiming to support the humeral head with a broad contact surface both proximally and distally. For the fibular strut, a 9 cm graft was prepared and fixated intramedullary, similar to a previous biomechanical study^2^ (Fig. 2, *B*). Fracture fixation was achieved using a locking plate (nine proximal and three shaft holes, PHILOS; Synthes, Oberdorf, Switzerland) with locking screws (Synthes, Oberdorf, Switzerland). All locking plates were fixated according to the manufacturer's guidelines. All plates were fixated with nine proximal locking screws, 1 diaphyseal cortical screw, and two diaphyseal locking screws to achieve maximum biomechanical stability.^11^ Screw lengths were assessed for all screws with a measuring tool provided by the manufacturer. An experienced upper limb surgeon (L.K.) performed fracture creation and graft preparation in all shoulders. Plate fixation was performed by L.K. and E.T.

### Biomechanical testing

**Figure 3:**
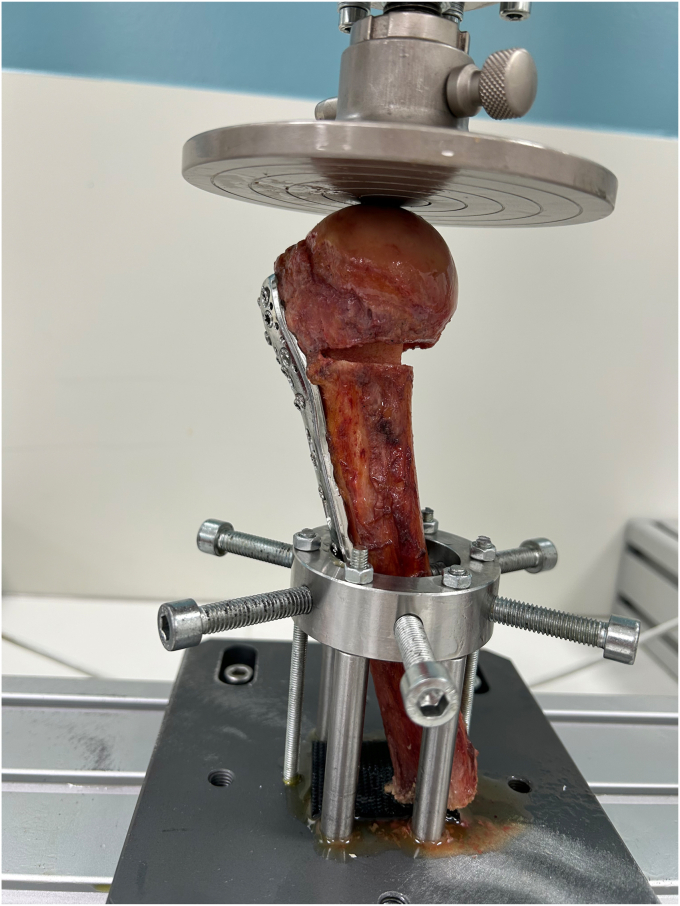
The mechanical test setup and fixation of the proximal humerus.

Biomechanical testing was performed using a Litem pneumatic modular frame vertical double-column testing machine with TC-Micro series load cell (L-TC-M-500; Litem, Ancona, Italy) (Fig. 3). This system enables applying axial loads to the humerus. The humerus was clamped in the machine using a custom mount at a 110° angle (Fig. 3). The specimens were first preloaded with 5 N to obtain the baseline measurements and then subjected to cyclic loading from 5 N to 532.6 N. The maximum force represents the maximum reaction force in an average male shoulder during 1000 compressive cycles at a frequency of 1 Hz as the highest load reduction and loss of fracture stabilization mostly occur within these cycles.^36^ The applied load was chosen according to a similar biomechanical study.^4^ After cyclic loading, an increasing axial load was applied at a rate of 10 N/s until failure or 1700 N, the maximal applicable load. Cyclic loading was used to simulate forces created during regular motion and activities, as postoperative varus deformation and screw protrusion mostly occur after early passive motion.^26^ Load-to-failure tests were subsequently performed to simulate forces in extreme conditions. The loss of humeral head height (HHH) of the construct was calculated by assessing the differences in actuator position before and after cyclic and failure testing. The number of cycles to failure, the maximum failure load, stiffness, and failure mode (bone or plate) were also assessed and compared between the two groups.

### Power analysis

Our sample size calculations were based on previous literature comparing fibular or femoral allografts with nonaugmented locking plate fixation, as no studies have compared the two augmentation techniques.^5^^,^^8^ Reported effect sizes were similar across both graft types. Power analysis (95% power, α = 0.05) indicated that nine humeri per group would be sufficient to detect significant differences when comparing fibular grafts to nonaugmented fixation, whereas ten humeri per group were required for the femoral graft comparison. Given our noninferiority design, a conservative sample size of ten humeri per group was chosen.

### Statistical analysis

Descriptive statistics of cadavers and outcomes are presented as numbers with percentages or as median with 25th and 75th percentiles (nonparametric data). All individual load-displacement graphs from the load-to-failure testing were assessed for linearity, and the stiffness of each construct was calculated. Failure was defined as stagnation of displacement while applied force was still increasing (eg, screw protrusion or loss of reduction due to humeral head failure) or a rapid increase of displacement combined with decreasing force (eg, complete construct failure) in the individual load-displacement graphs. To compare maximal load to failure, stiffness, and loss of HHH, a Mann-Whitney U test was used because of small sample sizes. *P* values <.05 were considered significant. Microsoft Excel 2016 (Microsoft Corporation, Redmond, Washington, USA) and R (R Core Team [2020]. R: A language and environment for statistical computing. R Foundation for Statistical Computing, Vienna, Austria) was used for statistical analyses.

## Results

### Specimens

Twenty-two human shoulders were tested in the study. Two constructs were excluded from the final analysis as they were extreme outliers (1 fibula and 1 femoral graft). The two constructs failed early during cyclic loading with complete obliteration of the humeral head, likely due to insufficient bone quality. The cortical fibular allograft group consisted of five right (50%) and five left shoulders (50%). The cancellous femoral head allograft was used in six right shoulders (60%) and four left shoulders (40%). There were no significant differences in cortex diameters across both groups (Table I).

**Table I:** Specimens and test results.

	Fibular graft (n = 10)	Femoral graft (n = 10)	*P* value
Cortex thickness, mm	3.00 (2.00-3.00)	3.00 (2.00-3.00)	.83
Shaft diameter, mm			
Medial - lateral	3.50 (3.20-3.88)	3.75 (3.55-3.90)	.44
Anterior - posterior	2.80 (2.50-3.00)	3.10 (2.65-3.38)	.22
Cyclic testing			
Loss of HHH, mm	2.00 (1.12-3.50)	2.75 (2.00-3.00)	.40
Load-to-failure testing			
Loss of HHH, mm	9.50 (7.88-15.50)	9.50 (8.50-10.90)	.68
Ultimate load, N∗	1624 (1524-1652)	1334 (1264-1499)	.26
Stiffness, N/mm	174.0 (106.0-186.0)	157.0 (134.0-192.0)	.97

*HHH*, humeral head height.

All results are presented as median with 25th and 75th percentiles.

∗ The ultimate load across both groups for the failed constructs (4 in fibula and 6 in hip).

### Cyclic loading and load-to-failure tests

**Figure 4:**
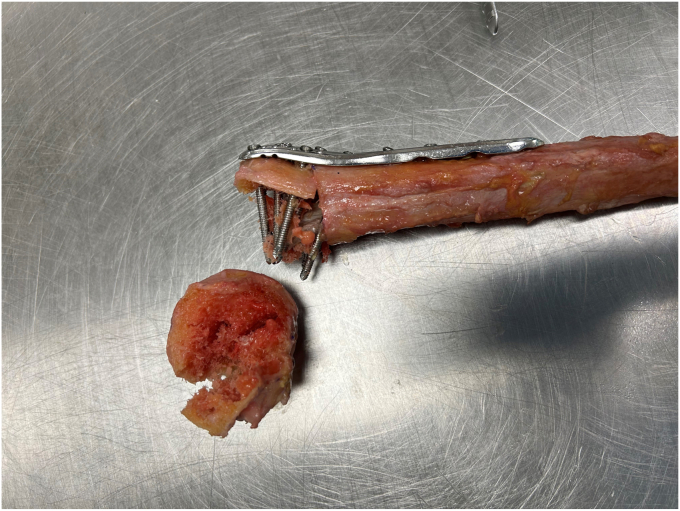
Example of a failed construct augmented with a fibular strut graft. The failure mode was a fractured humeral head.

Failure did not occur during cyclic loading in any of the constructs. After the cyclic tests, median loss of HHH was 2.0 (interquartile range [IQR], 1.0-2.0) mm in the fibular group and 2.75 (IQR, 2.0-3.0) mm in the femoral head group (*P* = .18). All constructs subsequently completed the load-to-failure tests. Four humeri augmented with fibular allografts (40.0%) and six augmented with femoral allografts (60.0%) failed. The mode of failure was a fractured humeral head in all failed constructs (Fig. 4). The median load to failure for the fibular group was 1624 (IQR, 1524-1652) N, while the femoral allografts failed at a median load of 1334 (IQR, 1264-1499) N (*P* = .26). The median stiffness of constructs with the fibular graft was 174 (IQR, 106-186) N/mm, and 157 (IQR, 134-192) N/mm for constructs augmented with the femoral head (*P* = .97). All results of biomechanical testing are depicted in Table I.

### Failed vs. intact constructs

Failed constructs were compared with intact constructs after load-to-failure tests for both groups. In the constructs augmented with the femoral grafts, cortex thickness (*P* = .05) and medial-lateral shaft diameter (*P* = .01) significantly differed. No other significant differences were found (Table II).

**Table II:** Failed vs. intact constructs.

	Fibular graft			Femoral graft		
	Failed (n = 4)	Intact (n = 6)	*P* value	Failed (n = 6)	Intact (n = 4)	*P* value
Cortex thickness, mm	2.50 (2.00-3.00)	3.00 (2.30-3.80)	.42	2.00 (2.00-2.80)	3.00 (3.00-3.30)	**.05**
Shaft diameter, mm						
Medial - lateral	3.25 (3.00-3.60)	3.50 (3.50-3.90)	.32	3.60 (3.20-3.70)	3.90 (3.90-4.10)	**.01**
Anterior - posterior	2.60 (2.50-2.80)	3.00 (2.60-3.00)	.33	2.80 (2.50-3.10)	3.50 (3.20-3.60)	.11
Cyclic testing						
Loss of HHH, mm	3.00 (1.60-5.90)	1.75 (1.10-2.00)	.52	3.00 (2.30-3.00)	2.30 (2.00-2.60)	.50
Load-to-failure testing						
Loss of HHH, mm	14.50 (11.20-17.60)	9.30 (7.90-9.50)	.39	9.50 (8.00-10.60)	9.50 (8.50-11.50)	.75
Stiffness, N/mm	114.0 (93.4-140.0)	184.0 (179-217.0)	.11	148.0 (134.0-163.0)	181.0 (151.0-200.0)	.75

*HHH*, humeral head height.

All results are presented as median with 25th and 75th percentiles. Bold *P* values indicate statistical significance (*P* ≤ .05).

## Discussion

This biomechanical study aimed to compare two augmentation techniques, a cortical fibular allograft and a cancellous femoral head allograft, for improving the stability of locking plate fixation in PHFs. During cyclic loading, a simulation of daily activities, none of the constructs in both groups failed with minimal loss of HHH. Furthermore, some constructs in both groups did not fail at the maximal applied force (1700 N), and the difference in stiffness between the two augmentation groups was not significant. This suggests that both constructs can withstand large amounts of forces and provide excellent stability in a PHF. Early failure of locking plate fixation usually occurs due to varus collapse and screw penetration through the humeral head.^27^ This is caused by a lack of bone stock and decreased medial stability due to comminution of the medial calcar after fractures, especially in osteoporotic bone.^14^^,^^27^^,^^28^ For those instances, the tested allografts may be a valuable option. They provide stability to the comminuted medial calcar and fill bone void for adequate locking screw fixation.^13^

Several studies have biomechanically compared the use of a cortical strut graft to nonaugmented plate fixation with the augmented group being superior in all studies.^2^^,^^7^^,^^15^^,^^23^ The augmented groups showed greater stability, a higher load of failure and a greater stiffness. The cancellous augmentation technique also showed a significantly greater stiffness compared to locking plate fixation alone. Dankl et al reported increased stiffness values up to five times when using hip allografts shaped similarly to our cancellous grafts.^8^ In our study, the cortical grafts showed greater stiffness (median 179 N/mm vs. 157 N/mm), but the difference was not significant. Moreover, the displacement at cyclic and load-to-failure testing was comparable, and ultimate loads in the failed constructs did not differ significantly. Furthermore, the median loss of HHH was less than 3 millimeters in both groups after cyclic loading (simulation of forces during regular motion and activities), a commonly used threshold for loss of reduction after plate fixation.^24^^,^^38^ When comparing failed constructs with intact constructs after load-to-failure testing, cortex diameter and medial-lateral shaft width were significantly greater in intact humeri augmented with cancellous femoral grafts. This may have resulted in lower stiffness and higher displacement in the failed constructs.

Apart from the evaluated biomechanical differences, the cortical fibular grafts have the advantage that shaping for optimal fit in the humerus is not necessary. The surgeon can choose the preferred length, and implantation is performed quickly. Shaping the cancellous femoral graft may take more time. First, the cortex needs to be removed, and subsequent shaping is needed to implant the graft into the humeral head and shaft. This may result in a longer operation duration of approximately five to ten minutes. However, one major pitfall of cortical strut allografts is the challenge they pose for revision surgery with arthroplasty once ingrowth and remodeling of the allograft has occurred.^9^^,^^21^^,^^22^ Removal of the cortical strut is a difficult and time-consuming procedure, and obliteration of the intramedullary humeral canal increases the risk of iatrogenic shaft fractures. While both augmentation techniques offer similar stability, the challenges posed by cortical fibular allografts may influence the choice for augmentation. Shi et al reported that a significant number, 8.2% of elderly patients treated with plate fixation require conversion to arthroplasty within 10 years.^32^ For patients with a higher likelihood of requiring future arthroplasty, a cancellous graft may be more beneficial, as it offers comparable mechanical stability and future revision surgery is less complicated. Additionally, in the Netherlands, costs for fibular grafts are higher than femoral grafts with a price difference of €650 in favor of the latter. Finally, in our experience, femoral grafts are readily available in most hospitals, whereas fibular grafts are often not.

Literature has shown that functional outcomes of fibular strut allografts compared to nonaugmented plate fixation are in favor of the former. Several systematic reviews showed significantly higher functional outcomes and similar or lower complication rates in patients with augmented plate fixation.^6^^,^^9^^,^^25^ Although clinical outcomes for cancellous hip allografts are scarce, Euler et al reported a median Constant score of 72 and 1 complication after a 2-year follow-up period.^10^ Future studies must compare functional outcomes, complications, and costs in a randomized controlled design. Moreover, long-term outcomes and differences in potential revision surgery need to be compared in order to explore clinical benefits.

### Limitations

Our results should be viewed in the light of several limitations. First, this is an in vitro biomechanical study. It is not possible to evaluate differences in potential bone healing that may occur after several months. Moreover, possible clinical advantages, such as less challenging revision surgery, cannot be derived from biomechanical analyses. Second, no information regarding characteristics of human specimens or bone density were available. The specimens are donated for science purposes, and records were not accessible, and we did not have the resources to determine bone density of our specimens. Therefore, we could not correct for potential confounding of bone quality, and this should be considered when interpreting our results, as subtle group differences could not be excluded. Nevertheless, cortex thickness and shaft diameters were measured and did not differ significantly between the fibular and femoral allograft groups. Cortical thickness has previously shown to be a reliable predictor of bone mineral density in the proximal humerus,^35^ and therefore the similarity between groups in our study suggests that bone quality may be comparable. Moreover, our maximal load to failure in the failed constructs was in a similar range as previous studies using a testing method comparable to ours.^2^^,^^16^ Third, our displacement measurements were not derived from image-based tracking but from actuator position. This limited us from determining the precise source of displacement within each construct. Fourth, a flat loading plate was used. This could result in differences in compression between specimens due to varying bone quality, potentially influencing displacement-based outcomes such as stiffness. However, all specimens were tested in a standardized fashion, and applied forces were identical for each construct. Furthermore, randomization of specimens and comparable cortex thickness and shaft diameters reduce the potential of variability of constructs between the two groups. Fifth, 1700 N was the maximal applicable load. Not every construct failed, and thus we could not determine the true maximal load to failure for some constructs. However, the authors believe that a load higher than 1700 N (ie, a load of 170 kg) is not representative of clinical situations. If constructs can withstand 1700 N, they will most certainly not fail when normal loads are applied. Sixth, only axial loads could be applied in this study. Although this method was previously used in other biomechanical studies for PHFs,^2^^,^^16^^,^^30^ only loss of HHH could be measured, and we could not simulate rotational forces created by the rotator cuff. Seventh, inherent in biomechanical studies, our sample size was relatively low. Nevertheless, we performed a power calculation, a conservative sample size was chosen, and the required specimens were included. Finally, all specimens were stripped of soft tissues. Consequently, supplemental fixation methods such as suture augmentation through the rotator cuff, potentially resulting in greater stability, were not accounted for. While this limits direct clinical extrapolation, the aim of this study was to compare fibular and femoral allografts, and our findings reflect differences in allograft rather than soft-tissue augmentation.

## Conclusion

There is no significant difference in maximum failure loads and stiffness between cortical fibular allograft augmentation and cancellous hip allograft augmentation for locking plate fixation in PHFs. Both techniques may prevent early screw protrusion and varus collapse. Cancellous femoral allografts may be advantageous considering costs, availability, and future revision surgery with arthroplasty when compared to cortical fibular allografts.

## Acknowledgment

The authors would like to acknowledge Synthes for providing all the plates, screws, and materials that were required for this study. The authors also thank the Arthroscopy & Arthroplasty Courses Utrecht for their help with collecting the specimens. Finally, they would like to thank J.T.C. Beekhuis, M.B.H. Rondhuis, and P. Nieuwenhuizen from the Department of Anatomy at UMC Utrecht for their practical help while carrying out this study.

## Disclaimers

Funding: This study was funded by the Science Committee of Tergooi MC. The funding was used to cover the costs of renting the testing machine, with the funds being transferred directly to the company providing the equipment.

Conflicts of interest: The authors, their immediate families, and any research foundations with which they are affiliated have not received any financial payments or other benefits from any commercial entity related to the subject of this article.

## References

[1] Amini M.H. (2019). Managing the endosteal fibula during arthroplasty for proximal humeral fracture sequelae. J Orthop Trauma.

[2] Bae J.H., Oh J.K., Chon C.S., Oh C.W., Hwang J.H., Yoon Y.C. (2011). The biomechanical performance of locking plate fixation with intramedullary fibular strut graft augmentation in the treatment of unstable fractures of the proximal humerus. J Bone Joint Surg Br.

[3] Barlow J.D., Logli A.L., Steinmann S.P., Sems S.A., Cross W.W., Yuan B.J. (2020). Locking plate fixation of proximal humerus fractures in patients older than 60 years continues to be associated with a high complication rate. J Shoulder Elbow Surg.

[4] Burke N.G., Kennedy J., Cousins G., Fitzpatrick D., Mullett H. (2014). Locking plate fixation with and without inferomedial screws for proximal humeral fractures: a biomechanical study. J Orthop Surg (Hong Kong).

[5] Chang H.H., Lim J.R., Lee K.H., An H., Yoon T.H., Chun Y.M. (2023). Author Correction: the biomechanical effect of fibular strut grafts on humeral surgical neck fractures with lateral wall comminution. Sci Rep.

[6] Cheng H.Y., Liang C.W., Wang J.H., Kuo Y.R., Ko P.Y., Chuang C.H. (2025). The effects of augmentation choices for locking plate fixation in proximal humerus fracture osteosynthesis: a systematic review and meta-analysis. J Orthop Traumatol.

[7] Chow R.M., Begum F., Beaupre L.A., Carey J.P., Adeeb S., Bouliane M.J. (2012). Proximal humeral fracture fixation: locking plate construct +/- intramedullary fibular allograft. J Shoulder Elbow Surg.

[8] Dankl L., Schmoelz W., Hoermann R., Euler S. (2022). Evaluation of mushroom-shaped allograft for unstable proximal humerus fractures. Arch Orthop Trauma Surg.

[9] Dasari S.P., Kerzner B., Fortier L.M., Rea P.M., Bodendorfer B.M., Chahla J. (2022). Improved outcomes for proximal humerus fracture open reduction internal fixation augmented with a fibular allograft in elderly patients: a systematic review and meta-analysis. J Shoulder Elbow Surg.

[10] Euler S.A., Hengg C., Wambacher M., Spiegl U.J., Kralinger F. (2015). Allogenic bone grafting for augmentation in two-part proximal humeral fracture fixation in a high-risk patient population. Arch Orthop Trauma Surg.

[11] Fletcher J.W.A., Windolf M., Richards R.G., Gueorguiev B., Varga P. (2019). Screw configuration in proximal humerus plating has a significant impact on fixation failure risk predicted by finite element models. J Shoulder Elbow Surg.

[12] Foruria A.M., Carrascal M.T., Revilla C., Munuera L., Sanchez-Sotelo J. (2010). Proximal humerus fracture rotational stability after fixation using a locking plate or a fixed-angle locked nail: the role of implant stiffness. Clin Biomech (Bristol).

[13] Gardner M.J., Boraiah S., Helfet D.L., Lorich D.G. (2008). Indirect medial reduction and strut support of proximal humerus fractures using an endosteal implant. J Orthop Trauma.

[14] Gardner M.J., Weil Y., Barker J.U., Kelly B.T., Helfet D.L., Lorich D.G. (2007). The importance of medial support in locked plating of proximal humerus fractures. J Orthop Trauma.

[15] Hsiao C.K., Tsai Y.J., Yen C.Y., Lee C.H., Yang T.Y., Tu Y.K. (2017). Intramedullary cortical bone strut improves the cyclic stability of osteoporotic proximal humeral fractures. BMC Musculoskelet Disord.

[16] Jang Y., Kim D. (2021). Biomechanical study of Proximal humeral fracture fixation: locking plate with medial support screw vs. locking plate with intramedullary fibular graft. Clin Biomech (Bristol).

[17] Kim D.S., Lee D.H., Chun Y.M., Shin S.J. (2018). Which additional augmented fixation procedure decreases surgical failure after proximal humeral fracture with medial comminution: fibular allograft or inferomedial screws?. J Shoulder Elbow Surg.

[18] Lecoultre Y., Beeres F.J.P., Link B.C., Pretz F., Tillmann F., Babst R. (2024). Cement augmentation for proximal humerus fractures: a meta-analysis of randomized trials and observational studies. Eur J Trauma Emerg Surg.

[19] Lee S.H., Han S.S., Yoo B.M., Kim J.W. (2019). Outcomes of locking plate fixation with fibular allograft augmentation for proximal humeral fractures in osteoporotic patients: comparison with locking plate fixation alone. Bone Joint J.

[20] Lee C.W., Shin S.J. (2009). Prognostic factors for unstable proximal humeral fractures treated with locking-plate fixation. J Shoulder Elbow Surg.

[21] Longo U.G., Gulotta L.V., De Salvatore S., Lalli A., Bandini B., Giannarelli D. (2024). Augmented versus non-augmented locking-plate fixation in proximal humeral fractures. Bone Joint J.

[22] Manzi J.E., Ruzbarsky J.J., Rauck R.C., Gulotta L.V., Dines J.S., Dines D.M. (2020). Failed proximal humerus osteosynthesis using intramedullary fibular strut allograft conversion to reverse shoulder arthroplasty. Tech Hand Up Extrem Surg.

[23] Mathison C., Chaudhary R., Beaupre L., Reynolds M., Adeeb S., Bouliane M. (2010). Biomechanical analysis of proximal humeral fixation using locking plate fixation with an intramedullary fibular allograft. Clin Biomech (Bristol).

[24] Neviaser A.S., Hettrich C.M., Beamer B.S., Dines J.S., Lorich D.G. (2011). Endosteal strut augment reduces complications associated with proximal humeral locking plates. Clin Orthop Relat Res.

[25] Nie W., Wang Z., Gu F., Xu S., Yue Y., Shao A. (2022). Effects of fibular strut augmentation for the open reduction and internal fixation of proximal humeral fractures: a systematic review and meta-analysis. J Orthop Surg Res.

[26] Osterhoff G., Baumgartner D., Favre P., Wanner G.A., Gerber H., Simmen H.P. (2011). Medial support by fibula bone graft in angular stable plate fixation of proximal humeral fractures: an in vitro study with synthetic bone. J Shoulder Elbow Surg.

[27] Owsley K.C., Gorczyca J.T. (2008). Fracture displacement and screw cutout after open reduction and locked plate fixation of proximal humeral fractures [corrected]. J Bone Joint Surg Am.

[28] Ring D. (2007). Current concepts in plate and screw fixation of osteoporotic proximal humerus fractures. Injury.

[29] Schliemann B., Siemoneit J., Theisen C., Kosters C., Weimann A., Raschke M.J. (2012). Complex fractures of the proximal humerus in the elderly--outcome and complications after locking plate fixation. Musculoskelet Surg.

[30] Seide K., Triebe J., Faschingbauer M., Schulz A.P., Puschel K., Mehrtens G. (2007). Locked vs. unlocked plate osteosynthesis of the proximal humerus - a biomechanical study. Clin Biomech (Bristol).

[31] Seok H.G., Park S.G. (2023). Dual-plate fixation for proximal humerus fractures with unstable medial column in patients with osteoporosis. J Orthop Trauma.

[32] Shi B.Y., Upfill-Brown A., Wu S.Y., Trikha R., Ahlquist S., Kremen T.J. (2023). Short-term outcomes and long-term implant survival after inpatient surgical management of geriatric proximal humerus fractures. J Shoulder Elb Arthroplast.

[33] Stone M.A., Namdari S. (2019). Surgical considerations in the treatment of osteoporotic proximal humerus fractures. Orthop Clin North Am.

[34] Sudkamp N., Bayer J., Hepp P., Voigt C., Oestern H., Kaab M. (2009). Open reduction and internal fixation of proximal humeral fractures with use of the locking proximal humerus plate. Results of a prospective, multicenter, observational study. J Bone Joint Surg Am.

[35] Tingart M.J., Apreleva M., von Stechow D., Zurakowski D., Warner J.J. (2003). The cortical thickness of the proximal humeral diaphysis predicts bone mineral density of the proximal humerus. J Bone Joint Surg Br.

[36] Wheeler D.L., Colville M.R. (1997). Biomechanical comparison of intramedullary and percutaneous pin fixation for proximal humeral fracture fixation. J Orthop Trauma.

[37] Wong J., Newman J.M., Gruson K.I. (2016). Outcomes of intramedullary nailing for acute proximal humerus fractures: a systematic review. J Orthop Traumatol.

[38] Zhao L., Qi Y.M., Yang L., Wang G.R., Zheng S.N., Wang Q. (2019). Comparison of the effects of proximal humeral internal locking system (PHILOS) alone and PHILOS combined with fibular allograft in the treatment of neer three- or four-part proximal humerus fractures in the elderly. Orthop Surg.

[39] Zhu Y., Lu Y., Shen J., Zhang J., Jiang C. (2011). Locking intramedullary nails and locking plates in the treatment of two-part proximal humeral surgical neck fractures: a prospective randomized trial with a minimum of three years of follow-up. J Bone Joint Surg Am.

